# Loss of insulin-like growth factor II imprinting is a hallmark associated with enhanced chemo/radiotherapy resistance in cancer stem cells

**DOI:** 10.18632/oncotarget.9784

**Published:** 2016-06-02

**Authors:** Xin Zhao, Xiaoliang Liu, Guanjun Wang, Xue Wen, Xiaoying Zhang, Andrew R. Hoffman, Wei Li, Ji-Fan Hu, Jiuwei Cui

**Affiliations:** ^1^ Cancer Center, The First Hospital of Jilin University, Changchun, Jilin 130021, China; ^2^ Stanford University Medical School, Palo Alto Veterans Institute for Research, Palo Alto, CA 94304, USA

**Keywords:** cancer stem cells, epigenetics, genomic imprinting, IGF2, intrachromosomal looping

## Abstract

Insulin-like growth factor II (*IGF2*) is maternally imprinted in most tissues, but the epigenetic regulation of the gene in cancer stem cells (CSCs) has not been defined. To study the epigenetic mechanisms underlying self-renewal, we isolated CSCs and non-CSCs from colon cancer (HT29, HRT18, HCT116), hepatoma (Hep3B), breast cancer (MCF7) and prostate cancer (ASPC) cell lines. In HT29 and HRT18 cells that show loss of *IGF2* imprinting (LOI), *IGF2* was biallelically expressed in the isolated CSCs. Surprisingly, we also found loss of *IGF2* imprinting in CSCs derived from cell lines HCT116 and ASPC that overall demonstrate maintenance of *IGF2* imprinting. Using chromatin conformation capture (3C), we found that intrachromosomal looping between the *IGF2* promoters and the imprinting control region (ICR) was abrogated in CSCs, in parallel with loss of *IGF2* imprinting in these CSCs. Loss of imprinting led to increased *IGF2* expression in CSCs, which have a higher rate of colony formation and greater resistance to chemotherapy and radiotherapy *in vitro*. These studies demonstrate that *IGF2* LOI is a common feature in CSCs, even when the stem cells are derived from a cell line in which the general population of cells maintain *IGF2* imprinting. This finding suggests that aberrant *IGF2* imprinting may be an intrinsic epigenetic control mechanism that enhances stemness, self-renewal and chemo/radiotherapy resistance in cancer stem cells.

## INTRODUCTION

The discovery of cancer stem cells (CSCs) is a landmark in cancer research that has broad potential applications for targeted tumor therapy [1, 2]. CSCs are a small subgroup of cells residing in a heterogeneous population of tumor cells. CSCs, which usually comprise <1% of all cells in a given tumor, are thought to drive tumor progression, invasion, migration, metastasis, and drug-resistance [3, 4]. Cancer stem cells have been isolated from many kinds of malignant tumors, including leukemia [5], breast cancer [6], colon cancer [7], hepatoma [8], melanoma [9], lung cancer [10], prostate cancer [11], pancreatic cancer [12], and ovarian cancer [13]. Conventional cancer therapies may eliminate most of the tumor mass, but a small population of CSCs with the potential to repopulate the tumor may escape destruction and survive the therapy [14, 15]. Since successful eradication of a malignancy may require the elimination of the CSCs, it would be ideal to find a therapy that can specifically target and kill CSCs.

The growth hormone/insulin-growth factor (IGF) axis plays an important role in regulating self-renewal of cancer stem cells [16, 17]. The IGF pathway is frequently activated in a variety of cancers [18–20]. By stimulating the PI3-K and MAPK cascade pathways, IGF-I and IGF-II promote cell proliferation and induce resistance to chemotherapeutic agents, radiation, and targeted therapies [21, 22]. *IGF2* is maternally imprinted in most normal tissues, with only the paternal allele being expressed. In many tumors, however, this imprinting is lost, leading to biallelic expression of the gene [23–25]. Over-production of the growth factor promotes the malignant behavior of tumor cells through enhanced cell growth and CSC self-renewal [26], and loss of *IGF2* imprinting (LOI) is associated with tumor initiation [27, 28]. Moreover, *IGF2*-overexpressing tumors frequently display loss of *PTEN*, and they are often highly proliferative, exhibiting strong staining for phospho-Akt [28, 29].

In order to study the role of *IGF2* in the maintenance of CSC characteristics, we isolated CSCs from six cancer cell lines and examined the allelic expression and epigenetic regulation of *IGF2*.

## RESULTS

### Characteristics of isolated CSCs

**Figure 1:**
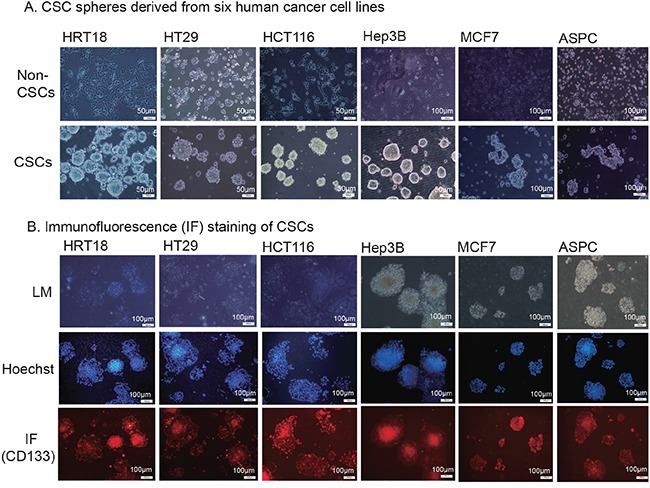
Isolation of cancer stem cells (CSCs) **A.** CSC spheres derived from six human cancer cell lines. Tumor cells were sorted by FACS to separate the CD133^−^ and CD133^+^ subpopulations. The CD133^−^ cells were cultured in RPMI 1640 or DMDM and were classified as the non-cancer stem cells (non-CSCs). The CD133^+^ cells were cultured in nonadhesive plates in cancer sphere medium (DMEM-F12 supplemented with 20 ng/ml bFGF, 20 ng/mL EGF, and 20 μl/ml B27). **B.** Immunofluorescence (IF) staining of CSCs. The sphere cells were treated with 4% paraformaldehyde, incubated with anti-CD133 antibody, and followed by the secondary antibody conjugated to fluorescent phycobiliproteins. Hoechst 33258 was used for nuclear counterstaining.

CSCs play an important role in tumor initiation, metastasis, and chemo/radiotherapy resistance [30]. To study the epigenetic mechanisms underlying CSC self-renewal, we isolated CSCs and non-CSCs from six solid tumor cell lines by flow cytometry analysis using an R-PE conjugated monoclonal antibody against CD133. The CD133-negative cells were collected and cultured in RPMI 1640 supplemented with 10% FBS. They maintained the same cell morphology as the parental cells (Figure 1A) and were subgrouped as “non-CSCs”. The CD133-positive cells were collected and cultured under stem cell suspension culture conditions to produce sphere-forming-like cells from single-dissociated cancer cells. Clusters of sphere-forming cells were successfully generated after 5-10 days of culture and were subgrouped as CSCs.

Self-renewal capacity is an essential feature of stem cells. We found that the enzymatically-dissociated sphere-forming-like cells maintained their spheroid morphology during serial passaging. The morphology of sphere-forming-like cells after 5 passages maintained the same characteristics as that of the first passage. Using immunofluorescence, we found that CSC marker CD133 continued to be expressed on the cell surface of HRT18, HT29, HCT116, Hep3B, MCF7 and ASPC CSCs (Figure 1B, bottom panel). More than 90% of the spheroid cells stained positive for CD133 (Supplementary Figure S1). The isolated CSCs also stained positive for the CSC marker, aldehyde dehydrogenase (ALDH, Supplementary Figure S2).

### Loss of IGF2 imprinting in CSCs

**Figure 2:**
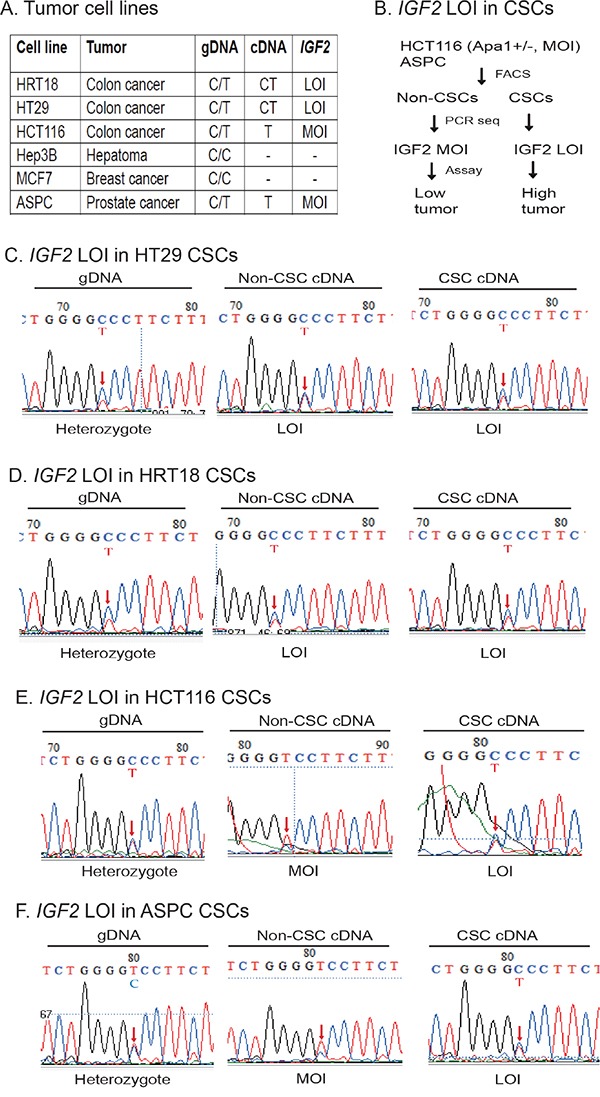
Differential loss of *IGF2* imprinting in CSCs **A.** Imprinting status in cancer cell lines. Using *ApaI* restriction enzyme typing and DNA sequencing of genomic DNA (gDNA), six human cancer cell lines were divided into informative (heterozygous C/T) and non-informative (homozygous C/C). By examining the expression of cDNA, HRT18 and HT29 were shown to demonstrate loss of *IGF2* imprinting (LOI). In contrast, HCT116 and ASPC were grouped as maintenance of *IGF2* imprinting (MOI). Hep3B and MCF7 were homozygous for the SNP and could not be used for imprinting analysis. gDNA: genomic DNA; cDNA: complementary DNA from reverse transcription. **B.** Differential *IGF2* imprinting between CSCs and non-CSCs. Two MOI tumor cells (HCT116 and ASPC) were separated into CSCs and non-CSCs. *IGF2* imprinting was examined by cDNA PCR sequencing. Restriction enzyme *ApaI* was used to genotype the *IGF2* alleles. **C.** Loss of *IGF2* imprinting in HT29 CSCs. Sequencing of genomic DNA shows the C/T heterozygosity. Red arrow: the site of the *ApaI* polymorphism. Note the biallelic expression of *IGF2* mRNA (LOI) in both non-CSCs and CSCs. **D.** Loss of *IGF2* imprinting in HRT18 CSCs. Both the non-CSCs and CSCs show loss of *IGF2* imprinting (LOI). **E.** Differential *IGF2* imprinting in HCT116 CSCs. In non-CSCs, only the T allele was detected, showing a typical imprinting pattern. In CSCs, however, both parental alleles were expressed (LOI). **F.** Differential *IGF2* imprinting in ASPC CSCs. Note the monoallelic expression of *IGF2* in non-CSCs, but the biallelic expression (LOI) in CSCs.

Among the six cancer cell lines, four were heterozygous (HRT18, HT29, HCT116, and ASPC) and thus informative for a SNP (C/T) located in *IGF2* exon 9 which can be used to distinguish the two parental alleles (Figure 2A). HRT18 and HT29 cell lines exhibited loss of *IGF2* imprinting (LOI), while HCT116 and ASPC maintained normal *IGF2* imprinting (MOI) [31–33]. We were particularly interested to determine if *IGF2* was differentially imprinted in CSCs as compared to non-CSCs (Figure 2B).

HT29 colon cancer cells were informative for the SNP, showing the presence of the “C” and “T” alleles in the genomic DNA (gDNA) (Figure 2C, left panel). As we previously reported [31–33], both the “C” and “T” alleles of *IGF2* mRNA transcripts are present in non-CSCs (middle panel), indicating loss of imprinting in this cancer cell line. In the CSCs derived from this cell line, *IGF2* was also biallelically expressed (right panel). Similarly, loss of *IGF2* imprinting was also detected in HRT18 non-CSCs and CSCs (Figure 2D).

On the other hand, we observed differential *IGF2* imprinting in HCT166 CSCs. In these cells, only the “T” allele was detected in the Non-CSC cells (Figure 2E, middle panel), indicating normal *IGF2* imprinting as previously reported [31–33]. However, in CSCs isolated from this cell line, we detected loss of *IGF2* imprinting, with both the C and the T alleles expressed (Figure 2E, right panel). These data demonstrate that *IGF2* imprinting can be differentially maintained between the non-CSC and CSC subpopulations in the same cell line.

ASPC is a pancreatic cancer cell line that was previously shown to maintain *IGF2* imprinting [31–33]. As expected, we found that *IGF2* was monoallelically expressed in non-CSCs (Figure 2F, middle panel). In CSCs, however, *IGF2* was biallelically expressed (right panel), suggesting that loss of *IGF2* imprinting is characteristic of CSCs in general, present even when stem cells were derived from a cell line that maintains *IGF2* imprinting.

### Chromosome conformation capture (3C)

Since maintenance of normal monoallelic expression of *IGF2* requires the presence of a CTCF-mediated long range intrachromosomal loop structure between the promoter and the imprinting control region (ICR), we then examined if there was a disruption of this intrachromosomal looping in the isolated CSCs.

**Figure 3:**
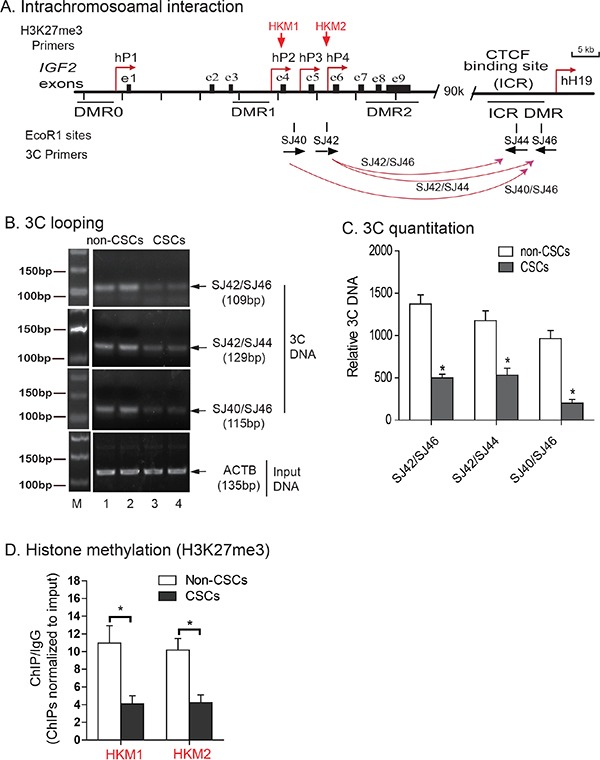
Abnormal intrachromosomal interactions between the ICR and *IGF2* promoters in CSCs **A.** Schematic diagram of *IGF2* intrachromosomal interactions. 3C primers: PCR primers used to detect intrachromosomal interactions; DMRs: Differentially methylated regions; P1-P4: human *IGF2* promoters; ICR: imprinting control region. The orientation and location of the 3C primers are shown by arrows under each EcoR1 site. **B.** The intrachromosomal interaction between the *IGF2* promoter and the CTCF-binding site in the ICR of HCT116 cells. M: 100 bp marker. The intrachromosomal interaction products were detected by 3C assay using primers in CTCF site combined with primers in *IGF2* promoters. Input DNA was used as the 3C control. Note the reduced 3C signals in CSCs. **C.** Quantitation of intrachromosomal interaction 3C products by quantitative PCR. All data shown are mean±SD from three independent. *p<0.01 as compared with non-CSCs. **D.** Histone methylation in the *IGF2* promoter. Levels of histone modifications in the *IGF2* promoter were measured by ChIP assay using antibodies specific for H3K27me3 in HCT116 non-CSCs and HCT116 CSCs. Normal rabbit IgG was used as a negative control. Precipitated DNA was subjected to qPCR. Bar graphs represent the ratio of precipitated DNA signals to IgG after normalization over the input. Error bars represent the standard error of the mean of three independent experiments. * p<0.05 between HCT116, non-CSCs and HCT116 CSCs.

We used the chromatin conformation capture technique (3C) [35] to detect intrachromosomal looping. Cells were fixed with 1% formaldehyde, digested with restriction enzyme *EcoRI*, and then ligated with T4 DNA ligase. Using this approach, the interacting chromatin complex DNAs are ligated and detected by PCR using the 3C primer sets located in the *IGF2* promoters (SJ38, SJ40, SJ42) and the ICR (SJ44, SJ46) (Figure 3A).

In the HCT116 non-CSCs that maintain normal *IGF2* imprinting, we detected three intrachromosomal interaction products: SJ42/SJ46 (109 bp), SJ42/SJ44 (129 bp), and SJ40/SJ46 (115 bp)(Figure 3B, lanes 1-2). In CSCs, however, only a weak intrachromosomal interaction signal was detected at all these three sites (lanes 3-4) in parallel with loss of *IGF2* imprinting. Quantitation of 3C products also showed a significantly lower intrachromosomal interaction signal in CSCs than that seen in non-CSCs (Figure 3C, p<0.01). These data suggest that the loss of this intrachromosomal interaction is associated with *IGF2* LOI [32, 33].

We then focused on *IGF2* promoter suppression by histone H3K27 methylation to determine whether this epigenetic suppressive mark was altered in CSCs in an association with loss of imprinting. Using chromatin immunoprecipitation (ChIP), we examined H3K27 methylation in the *IGF2* promoters. We observed a significant decrease in *IGF2* promoter H3K27 methylation in HCT116 CSCs as compared with the non-CSCs (Figure 3D), suggesting that H3K27 methylation is needed for *IGF2* imprinting.

### Upregulation of *IGF2* in CSCs

**Figure 4:**
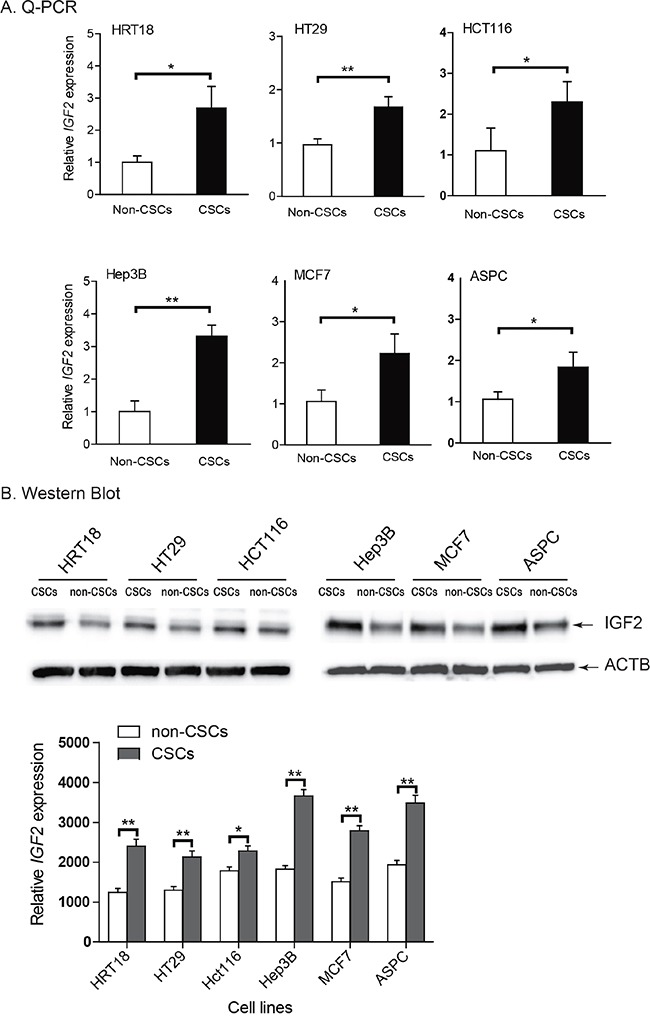
Upregulation of *IGF2* in CSCs **A.** Differential expression of *IGF2* between the non-CSCs and CSCs as measured by quantitative RT-PCR. Note the upregulated *IGF2* in CSCs as compared with that in non-CSCs. **B.** IGF*-*II protein as quantitated by Western blot. Protein expression was measured by imaging system Quantity One. All data shown are mean±SD from three independent experiments. * p<0.05, ** p<0.01 between the two groups.

Loss of imprinting can be associated with overexpression of the gene [36]. *IGF2* was significantly upregulated in HCT116 and ASPC CSCs, where there was loss of *IGF2* imprinting, compared to their non-CSC counterparts that maintain *IGF2* imprinting (Figure 4A). Increased *IGF2* expression was also observed in CSCs derived from HRT18 and HT29, in which there is *IGF2* LOI, as well as from Hep3B and MCF7 cell lines, which are homozygous at the SNP and are thus of unknown imprinting status. Using Western blotting, we also confirmed that *IGF2* was significantly upregulated in CSCs as compared with their non-CSC counterparts (Figure 4B, p<0.01).

### Enhanced tumor clonogenic activity in CSCs

**Figure 5:**
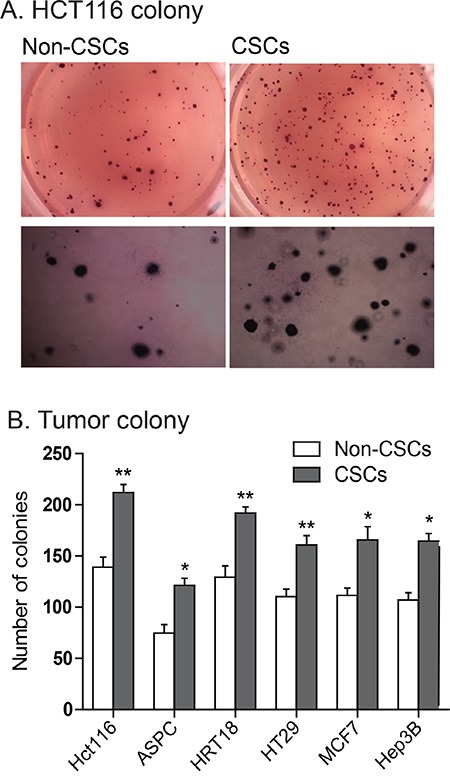
Enhanced ability to form tumor colonies in *IGF2*-upregulated CSCs **A.** Representative images of colonies of HCT116 cells stained with MTT in soft agar. **B.** Quantitation of tumor colonies in CSCs. Colonies were counted under microscopy and the results were presented as colonies per 500 cells. *P<0.05, **P<0.01 as compared with the non-CSCs. The results are expressed as the mean±standard deviation of three independent experiments.

A characteristic hallmark of CSCs is the capacity for self-renewal. The growth hormone/IGF axis plays an important role in regulating self-renewal of cancer stem cells [16, 17, 37, 38]. We used a clonogenic assay to examine the ability of a single cell to form a colony from CSCs that exhibited *IGF2*-upregulation. After incubation in soft agar for 13 days, colonies formed from both non-CSCs and CSCs, indicating their capacity for anchorage-independent growth. Figure 5A shows a typical tumor colony derived from HCT116 non-CSCs (MOI) and CSCs (LOI), showing that the CSCs formed more colonies. Similarly, we found that CSCs with *IGF2* upregulation exhibited a significantly greater ability to form colonies than did the non-CSC cells in all cell lines tested (Figure 5B, p<0.05).

### The IGF2-upregulated CSCs are resistant to chemo/radiotherapies

**Figure 6:**
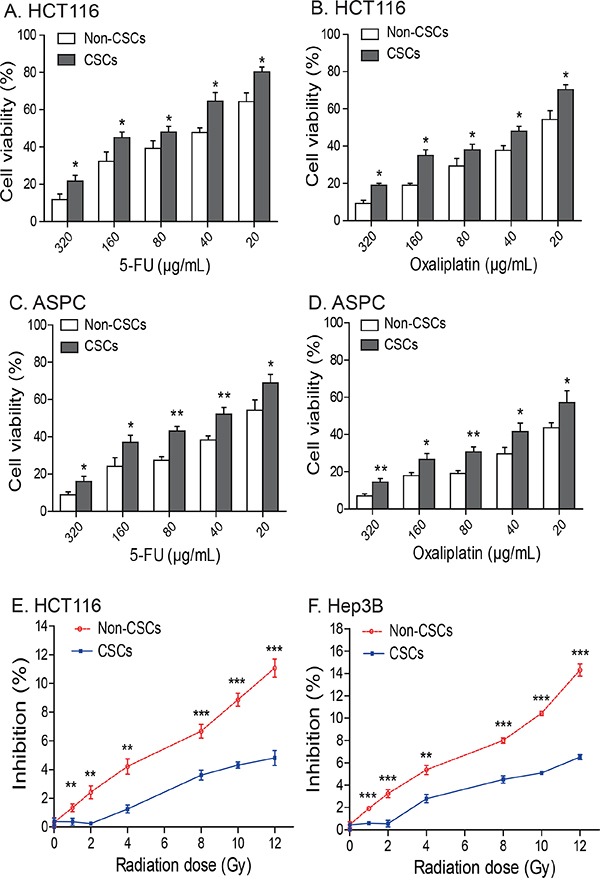
Chemotherapy and radiotherapy-resistance in CSCs **A-D.** *IGF2*-upregulated CSCs isolated from HCT16 and ASPC cells are resistant to treatment with 5-FU and oxalipatin. * *p*<0.05, ** *p*<0.01 as compared to the non-CSCs. The results are expressed as the mean ± standard deviation of three independent experiments. **E-F.** The *IGF2*-upregulated CSCs from HCT116 and Hep3B cells are resistant to radiotherapy. Cells were irradiated with 1-12Gy radiation and evaluated by WST-1 cell proliferation assays. ** *p*<0.01, *** *p*<0.001 as compared to the non-CSCs.

We then compared drug-sensitivity between the *IGF2* MOI non-CSCs and the *IGF2* LOI CSCs. Cells were treated with 5-fluorouracil (5-FU) and oxaliplatin, respectively, for 48 hours and were then assessed for cell viability. We found that HCT116 CSCs that had *IGF2* LOI were significantly more resistant to both 5-FU and oxaliplatin than were non-CSCs that exhibited normal *IGF2* imprinting (Figures 6A-6B, p<0.05). Similarly, greater resistance to 5-FU and oxaliplatin was also observed in ASPC CSCs with *IGF2* LOI than in the non-CSCs with *IGF2* MOI (Figures 6C-6D, p<0.05).

We also examined radiotherapy-resistance in two CSCs (HCT116 and Hep3B) with upregulated *IGF2* expression. In HCT116 non-CSCs that expressed *IGF2* monoallelically, there was an apparent dose-dependent inhibition of cell growth for all radiation doses. In CSCs that showed biallelic expression of *IGF2*, however, there was a much less inhibitory effect when the cells were exposed to <4 Gy of radiation. When exposure was > 4 Gy, CSCs still exhibited greater radiation-resistance than did the non-CSCs (Figure 6E). A similar radiation-resistant pattern was also observed in Hep3B CSCs (Figure 6F).

### IGF2 is critical to maintain the features of CSCs

**Figure 7:**
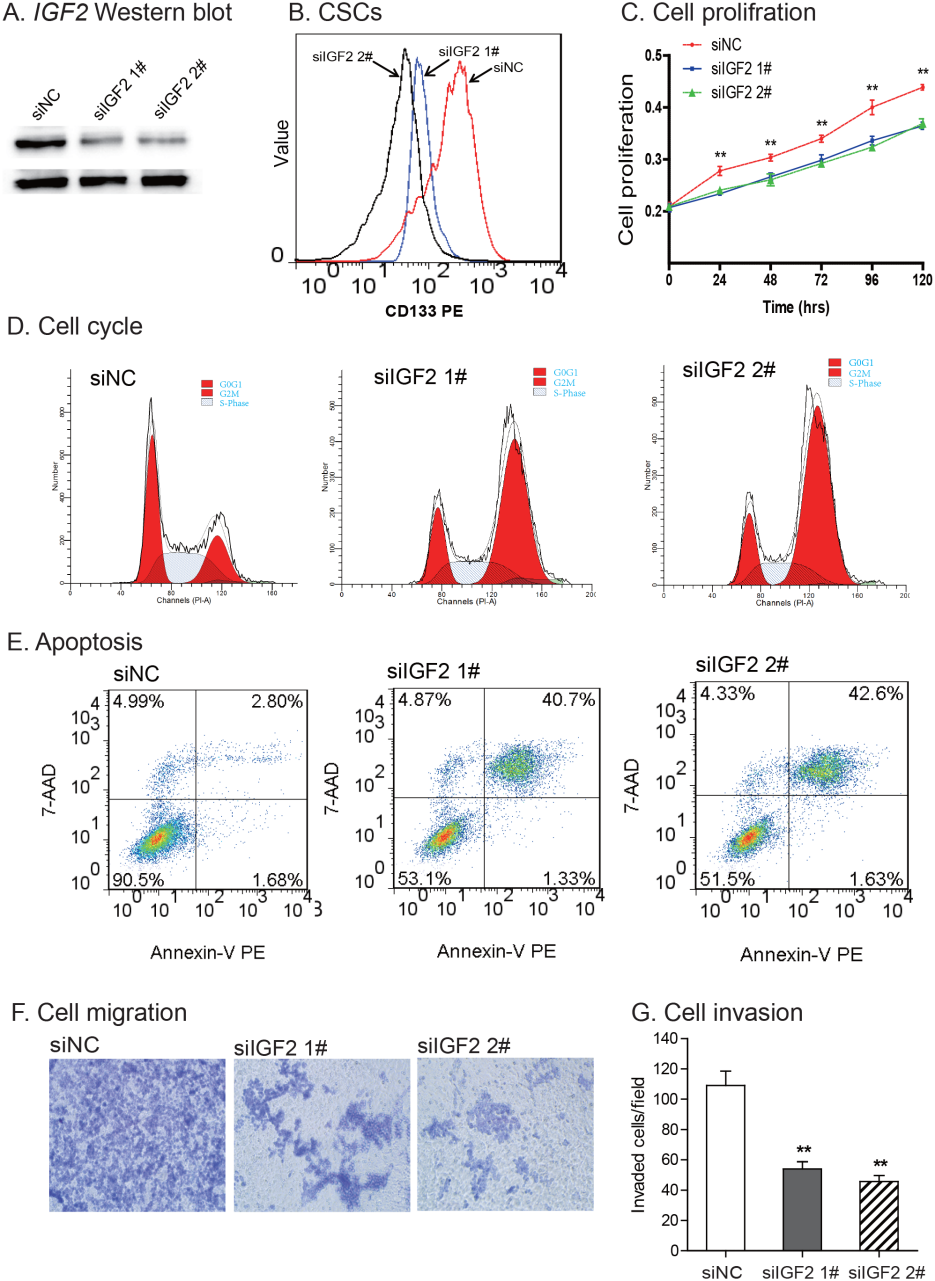
Knockdown of *IGF2* **A.** Knockdown of *IGF2* by siRNAs (siIGF2 1#, siIGF2 2#). siNC: negative control siRNA. The abundance of IGF-II protein was quantitated by Western blot. **B.** *IGF2* knockdown reduced CD133-positive cells. After siRNA treatment, CD133^+^ cells were analyzed by FACS. **C.** Reduced cell proliferation in *IGF2*-knockdown CSCs. **P<0.01 vs. control cells (siNC). **D.** Cell cycle analysis after interference of *IGF2* expression. After treatment with the control siRNA (siNC) and *IGF2* siRNA (siIGF2 1# and siIGF2 2#), cell cycle was analyzed by FACS. **E.** Apoptosis as measured by FITC Annexin V-FACS assay. **F.** Cell migration. *IGF2* knockdown reduced cell migration in HCT116 CSCs. **G.** Cell invasion. Cells that invaded through the collagen-coated membrane of the transwell were counted. All data shown are mean±SEM from three independent experiments. *p < 0.05 as compared with control cells (siNC).

To further examine the role of *IGF2* in CSCs, we used two siRNAs (siIGF2 1# and siIGF2 2#) to knock down *IGF2* in HCT116 CSCs (Figure 7A). *IGF2* knockdown significantly reduced the CD133^+^ cell population in HCT116 CSCs (Figure 7B), suggesting that *IGF2* is a critical growth factor in maintaining self-renewal of CSCs in colorectal cancer cells.

After *IGF2* knockdown, we also noticed a time-dependent inhibition of cell proliferation (Figure 7C). Using flow cytometry, we found that *IGF2*-knockdown induced an increased percentage of cells in G2/M-phase (23.46% in siNC vs 60.14% in siIGF2 1# and 67.08% in siIGF2 2#), in parallel with a decrease in G0/G1 phase (40.77% in siNC vs 17.73% in siIGF2 1# and 14.94% in siIGF2 2#)(Figure 7D).

Following *IGF2* knockdown, there was an increase in cell apoptosis in HCT116 *CSCs* (2.80% in siNC vs 40.7% in siIGF2 1# and 42.6% in siIGF2 2#)(Figure 7E). In addition, *IGF2*-knockdown in CSCs led to a reduction in cell migration (Figure 7F), invasion (Figure 7G), and resistance to chemo/radiotherapies (Supplementary Figure S3).

## DISCUSSION

*IGF2* is often overexpressed in a variety of human malignancies, partially due to the loss of genomic imprinting. However, the role of *IGF2* epigenetic regulation in cancer stem cells has not been elucidated. By examining allelic expression using an *IGF2* exon 9 SNP, we have shown that *IGF2* imprinting is lost in CSCs, including CSCs derived from two cancer cell lines in which *IGF2* is monoallelically expressed. This biallelic expression pattern is associated with upregulation of *IGF2* mRNA and IGF-II protein, leading to tumor growth and CSC self-renewal. CSCs exhibit significantly higher capacity to form tumor spheres than do the non-CSCs. Knockdown of *IGF2* led to a reduction of CD133-positive cells and decreased tumorigenesis, suggesting a role for loss of *IGF2* imprinting in the maintenance of stem cell renewal.

Specific biallelic expression of *IGF2* in CSCs suggests that *IGF2* imprinting can be a differential trait in a cell line with a mixed cell population. *IGF2* LOI is maintained in the CSC niche during cell passaging. In previous studies, allelic expression was examined in RNA extracted from the totality of the cells, and not specifically from the very small population of cancer stem cells. Similarly, *IGF2* LOI has been reported in a variety of human cancers at widely varying rates, including Wilms' tumor, colorectal cancer, hepatoma, lung cancer, ovarian cancer, bladder cancer, leiomyosarcoma, and esophagus cancer [39]. Because all of those published studies examined the status of *IGF2* imprinting in whole cancer tissues or cell lines, rather than in purified CSCs, it is likely that the true incidence of *IGF2* LOI, particularly in CSCs, has been greatly underestimated. It is also interesting to note that the increased *IGF2* expression was also observed in HRT18 and HT29 cell lines, where both the non-CSCs and CSCs exhibit loss of *IGF2* imprinting. Thus, aberrant imprinting is not the only factor that determines the abundance of *IGF2*. Future studies are needed to track *IGF2* imprinting at a single daughter cell level and identify other pathway factors that upregulate *IGF2* in CSCs.

An important characteristic of cancer stem cells and other stem cells is their asymmetrical division, resulting in two unequal daughter cells. Due to this unsymmetrical division, only one of the daughter cells resembles the parent stem cell and can maintain stemness. Currently, it is not clear if *IGF2* imprinting is inherited in this unsymmetrical division.

Loss of *IGF2* imprinting in peripheral blood leukocytes may provide a potential biomarker for diagnosing individuals who have a high risk of colorectal cancer [27, 40]. Tumor phenotype in Apc+/Min mice can be modified simply by altering the *IGF2* epigenotype [41]. By introducing DNA hypermethylation in the gene promoter with methylated hairpin oligonucleotides, we have previously demonstrated that silencing *IGF2* reduced the growth of implanted human hepatocarcinomas and prolonged lifespan in an animal model [42, 43]. Future studies are needed to examine if *IGF2* is also aberrantly imprinted in CSCs isolated from clinical tumor samples.

In summary, our data demonstrate for the first time that loss of *IGF2* imprinting is present in CSCs, even when the imprinting is maintained in the Non-CSC cells derived from the same population of cells. CSC *IGF2* LOI may underlie the stem cell characteristics of self-renewal and resistance to chemo/radiotherapies. We have shown that we can specifically target tumor cells in which *IGF2* is biallelically expressed [31, 44], and such targeting might prove to be a magic bullet to specifically kill the CSCs within a tumor.

## MATERIALS AND METHODS

### Cell lines and cell culture

Six cancer cell lines that exhibit distinct *IGF2* imprinting patterns were purchased for this study from American Type Culture Collection (ATCC, VA): HT29 (colon cancer), HRT-18 (colon cancer), HCT-116 (colon cancer), Hep3B (liver cancer), MCF-7 (breast cancer), and ASPC (pancreatic cancer). HT29 and HRT18 were shown to have loss of *IGF2* imprinting, while HCT116 and ASPC were shown to maintain normal *IGF2* imprinting [32, 34]. Hep3B and MCF7 were not informative at a variety of polymorphic sites, so we were unable to identify their imprinting status.

HT29, HRT-18, HCT- 116, and ASPC cell lines were maintained in RPMI 1640. Hep-3B and MCF-7 cells were cultured in Dulbecco's modified Eagle's medium (DMEM) media (HyClone, UT). Both media were supplemented with 10% fetal bovine serum (FBS), penicillin (100 U/ml), and streptomycin (100 μg/ml). Exponentially growing cells were collected by trypsin-EDTA for imprinting analyses.

### Isolation of CSCs

As previously reported [45–47], expression of the CSC marker CD133 was used to isolate CSCs. Briefly, nonspecific binding of cell membranes was blocked by incubating with 5% BSA for 30 min at room temperature. Cells were then incubated with CD133 antibody (MACS, Miltenyi Biotec, Germany) for 30 min before being subjected to flow cytometry analysis (FACSAria, BD, CA). Cells with the highest and the lowest fluorescence levels of CD133 were sorted and collected and they were designated as CD133^+^ cells (CSCs) and CD133^−^ cells (non-CSCs). CSCs were expanded in CSC spheres as in the following section for analyses.

### Flow cytometry analysis of CSCs

Cells were stained with the conjugated fluorescent antibody for 30 min at 4°C. Anti-CD133-PE was purchased from Miltenyi Biotec. After being washed with phosphate-buffered saline (PBS), ten thousand events per sample were acquired and the number of CD133^+^ cells was determined by flow cytometry.

### CSC sphere culturing

To evaluate the self-renewal characteristics of the isolated CSCs, the CD133^+^ cells were reseeded and cultured in CSC-culturing medium consisting of serum-free DMEM-F12 (HyClone, UT), supplemented with 20 ng/mL basic fibroblast growth factor (bFGF), 20 ng/mL epidermal growth factor (EGF)(Peprotech, NJ), and 20 μl/ml B27 supplement (Invitrogen, CA) in a ultra-low attachment 6-well plate (Corning, CA) at a density of < 5000 cells/well. CD133^−^ cells were cultured as the attached cells in RPMI 1640 or EMEM medium supplemented with 10% FBS, and they served as a control.

CSC spheres were defined as cell colonies with a diameter >200 μm and area >50% showing three-dimensional structure and blurred cell margins. Spheres began to form at day 4-5 of culturing. When the sphere-forming cells reached a diameter of >200 μm in the suspension culture, the cells were collected and dissociated with Accutase (Millipore/Upstate, CA) at 37°C for 3-5 min. After centrifugation, cells were washed with two volumes of PBS to inactivate the enzyme. Single CSCs were resuspended in CSC-culturing medium, and seeded for generation of secondary spheres. Spheres were observed under a microscope and images were photographed under a phase contrast fluorescence microscope (Nikon, ECLIPSE 80i, Japan).

After 4-5 passages, CSC spheres were collected and used for assays in this study, including *IGF2* imprinting, epigenetic mechanisms, cell migration and invasion, and chemo/radiotherapies.

### Immunofluorescence analysis

Cells were fixed in PBS containing 4% paraformaldehyde (NJ-reagent, Nanjing, China) for 30 min and permeabilized with phosphate-buffered saline containing 0.1% Triton X-100 (NJ-reagent) for 10 min. The samples were incubated with anti-CD133 antibody (1:100; Santa Cruz Biotechnology, Santa Cruz, CA, USA), followed by the secondary antibody conjugated to Cy3 NHS Ester goat anti-rabbit immunoglobulin (1:100; Beyotime, Shanghai, China).

The isolated CSCs was also stained with anti-ALDH antibody (1:100; Becton, Dickinson and Company, NJ, USA), followed by the secondary antibody conjugated to Alexa-Fluor 488-conjugated goat anti-rabbit IgG (1:200; Beyotime, Shanghai, China). Hoechst 33258 (Beyotime, Shanghai, China) was used for nuclear counterstaining.

### Allelic expression of IGF2

Tumor cell lines were first genotyped for the heterozygosity of SNPs in *IGF2* exon 9 mRNA. Tumor cells that were informative for SNP sites were used for *IGF2* imprinting studies (Supplementary Table S1). *IGF2* transcripts were amplified by RT–PCR (40 cycles of 96°C for 20 s, 58°C for 20 s and 72°C for 20 s, followed by a 3-min extension at 72°C) using primers specific for two polymorphic restriction enzymes (*ApaI*, *AluI*) in the last exon of human *IGF2* [32]. To determine the status of *IGF2* imprinting, the amplified products were sequenced by MyGenostics (Beijing, China). Cells that maintain normal imprinting (MOI) express a single parental allele, while the LOI showed biallelic expression of *IGF2*. PCR primers used for *IGF2* imprinting are listed in Supplementary Table S1.

### Chromosome conformation capture (3C)

The 3C assay was performed as described previously [32, 35, 48, 49]. In brief, HCT116 cells were cross-linked with 2% formaldehyde and lysed with cell lysis buffer (10 mM Tris, pH 8.0, 10 mM NaCl, 0.2% NP-40, and protease inhibitors). Nuclei were suspended in 1×restriction enzyme buffer in the presence of 0.3% SDS, and incubated at 37°C for 1 h. Triton X-100 was then added to a final concentration of 1.8% to sequester the SDS. An aliquot of nuclei (2×10^6^) was digested with 800 U of restriction enzyme EcoRI at 37°C overnight. After stopping the reaction by adding 1.6% SDS and incubating the mixture at 65°C for 20 min, chromatin DNA was diluted with ligation reaction buffer, and 2 μg DNA was ligated with 4,000 U T4 DNA ligase (New England Biolabs, MA) at 16°C for 4 h (final DNA concentration of 2.5 μg/ml). After treatment with 10 mg/ml proteinase K at 65°C overnight to reverse cross-links and with 0.4μg/ml RNase A for 30 min at 37°C, DNA was extracted with phenol-chloroform and ethanol precipitated. The ligated DNA products were detected by PCR amplification and quantitative PCR. PCR primers used for 3C assay are listed in Supplementary Table S1.

### Histone methylation by chromatin immunoprecipitation (ChIP) assay

ChIP assay was used to quantitate the status of histone modification in the isolated CSCs, following the manufacturer's protocol (Upstate Biotechnology, Lake Placid, NY). Specific anti-trimethyl-histone H3 (Lys27) antibody (Merck Millipore, Darmstadt, Germany) was used to determine histone methylation and the normal isotype-matched IgG from the same species was used as negative control. Precipitated DNA was subjected to qPCR. ChIP PCR data are expressed as fold enrichment versus IgG chromatin input using comparative ΔΔCt analysis [32, 33].

### Soft-agar clonogenic assay

The clonogenic assay was performed using the method previously reported [50, 51] to examine the tumorigenicity of the cultured CSCs. Briefly, 0.5% and 0.25% soft agarose (Sigma, MO) were prepared with sterile H_2_O and stored at 4°C. DMEM culture medium containing 10% FBS was kept in a 37°C water bath. 60μl of 0.5% agarose and 540μl DMEM were mixed and layered onto 24-well plates as the base agar. CSCs were digested, centrifuged and resuspended in DMEM medium to form a single cell suspension. The top agar cell suspension (2×10^3^ cells/ml) was prepared by mixing cells with 0.25 ml DMEM and 0.25 ml 0.5% agarose, and then adding this onto the base agar. The plates were incubated at 37°C, 5% CO_2_ until visible colonies (N>50/group) were observed. After ~2 weeks, colonies were visualized by staining with 5 mg/ml MTT for 3 hr. Cloning forming efficiency (CFE) was calculated by the number of colony formations/the number of inoculated cells×100%.

### Chemotherapy sensitivity assays

The chemo-resistance of tumor sphere cells was assessed using WST-1 cell proliferation assays as previously reported [52]. Briefly, 1.25×10^4^ cells per well were seeded in 96-well plates in 200 μl culture medium (three wells per group). After 24 hr culture, cells were treated with various concentrations (0, 20, 40, 80, 160, 320 μg/ml) of 5-FU and oxaliplatin, respectively, for another 48 hrs. Cell proliferation was evaluated by adding 20 μl WST-1 reagent to each well. The reaction proceeded for 3 h at 37°C with 5% CO_2_. The absorbance of the samples at 450 nm was measured using a microplate reader. The effects of 5-FU and oxaliplatin on the viability of tumor sphere cells were expressed as the cell viability (%) = A450 of treated cells/A450 of control cells×100%. Three independent experiments were performed.

### Radiotherapy resistance assay

Tumor sphere cells were dissociated by trypsinization and were seeded at 2.5×10^4^ cells per well in 200μL medium in 96-well plates. The cells were exposed to various doses of radiation using an X-ray machine at room temperature. The sham-irradiated (control) cells were treated similarly but were not irradiated. Cells were irradiated by a Philips deep X-ray machine with 200 kVp, 10 mA and filters of 0.5 mm copper plus 1.0 mm aluminum. The dose rate was 1Gy/min X rays. Radiation doses were 1, 2, 4, 8, 10, and 12Gy. Immediately after irradiation or sham irradiation, cells were returned to the same culturing conditions.

### DNA extraction

Genomic DNA from cultured cells was extracted using the Cell/tissue genome DNA Extraction Kit (Generay Biotechnology, China) according to the manufacturer's protocol. DNA was dissolved in a final volume of 80 μL of buffer and quantified using a NanoVue spectrophotometer (GE, USA).

### Reverse transcription

Total RNA was extracted from the tumor cells using Trizol reagent (Invitrogen, CA). To eliminate residual genomic DNA, total RNAs (or total nucleic acids) were treated with DNase I (2 units/1mg of RNA)(Takara, Japan) for 50 min. Reverse transcription (RT) was performed with a cDNA Reverse Transcription Kit (Invitrogen).

Quantitative real-time PCR (Q-PCR) was performed using Faststart Universal SYBR Green Master (Roche). The Q-PCR assays were run in triplicate on 96-well plates using the ABI Prism 7900HT sequence detector, following the ABI protocol. Thermal cycling was performed by an initial denaturing step at 95°C for 10 min followed by 40cycles of denaturation at 95°C for 10 sec, 60°C for 45 sec forannealing and data collection. At the end of the Q-PCR, a melting curve analysis was used to confirm the homogeneity of all Q-PCR products. Relative transcript levels of each gene were calculated using the delta-delta Ct method, using a housekeeping gene (ACTB) as the reference gene. PCR primers used for ACTB and *IGF2* gene expression and are listed in Supplementary Table S1.

### Western blotting

Cells were lysed with immunoprecipitation assay buffer (1% Nonidet P-40, 50 mM Tris-HCl, pH 7.4, 150 mM NaCl, 1% sodium deoxycholate, 0.1% SDS, plus protease inhibitor cocktail and 1 mM phenylmethylsulfonyl fluoride). Proteins were separated by SDS-PAGE and analyzed by Western blotting. Antibodies to IGF-II and ACTB were obtained from Santa Cruz Biotechnology (Santa Cruz Biotechnology, CA, USA). IGF-II protein expression of was measured by imaging system Quantity One.

### Knockdown of IGF2 by RNA interference

*IGF2*-specific siRNAs were purchased from Life Technologies Corporation (CA, USA). siIGF2 1#: sence:5′-GAGGAGUGCUGUUUCCGCAtt-3′, antisense: 5′- UGC GGAAACAGCACUCCUCaa-3′; siIGF2#2: 5′-ACAACCCUCUUAAAACUAAtt-3′, antisense: 5′-UUAGUUUUAAGAGGGUUGUtg-3′. The control siRNA (siNC) against photinus pyralis luciferase gene (Invitrogen, CA, USA) was 5′-GGAUUUC GAGUCGUCUUAAUGUAUA-3′. RNAiMAX transfection reagent was used for transient transfection following manufacturer's protocol (Invitrogen, CA, USA).

### Cell migration and invasion assay

Cell migration and invasion assays were carried out using Transwell membrane filters inserted in 24-well tissue culture plates (6.5-mm diameter, 8-μm pore size)(Corning, MA) as previously described [52]. For the migration assay, 4 × 10^4^ cells suspended in serum-free medium were seeded on the top chamber of transwell filters. Serum containing medium was added to the bottom chamber and incubated for 16 h at 37°C. The non-migrating cells were removed by wiping the upper side of the filter, and the migrated cells on the bottom side of the filter were fixed with 4% formaldehyde and stained with crystal purple.

A similar protocol was used for the invasion assay, except that cells were seeded in Transwell chambers coated with 0.5μg/μl type I collagen (Invitrogen, CA, USA) [56]. Each assay represents the average of three independent experiment.

### Cell cycle analysis by flow cytometry

The cells were washed with PBS twice, pelleted and fixed with cold 70% ethyl alcohol for at least 30 minutes. After being washed twice with cold PBS, cells were incubated with 200 μg/ml RNaseA for 30 minutes at 37°C. The cells were stained with 100 μg/ml propidium iodide for 30 minutes at room temperature. The samples were immediately analyzed by flow cytometry. Cell cycle phase distribution was determined using Cell Quest Pro software.

### Statistical analysis

All experiments were performed in triplicate, and the data are expressed as mean ± SD. The comparative *C*T method was applied in the quantitative real-time RT-PCR assay according to the delta-delta *C*T method [31, 34]. The data were analyzed with one-way analysis of variance (ANOVA), and results were considered statistically significant at P ≤ 0.05.

## SUPPLEMENTARY FIGURES AND TABLE


