# Supplementary material for: Loss of insulin-like growth factor II imprinting is a hallmark associated with enhanced chemo/radiotherapy resistance in cancer stem cells
Source: Oncotarget. 2016 Jun 2;7(32):51349–64. doi: 10.18632/oncotarget.9784 (PMC5239480; doi:10.18632/oncotarget.9784)
Supplement: Supplementary file 1 [file oncotarget-07-51349-s001.pdf]

Loss of insulin-like growth factor II imprinting is a hallmark associated with enhanced chemo/radiotherapy resistance in cancer stem cells

SUPPLEMENTARY FIGURES AND TABLE

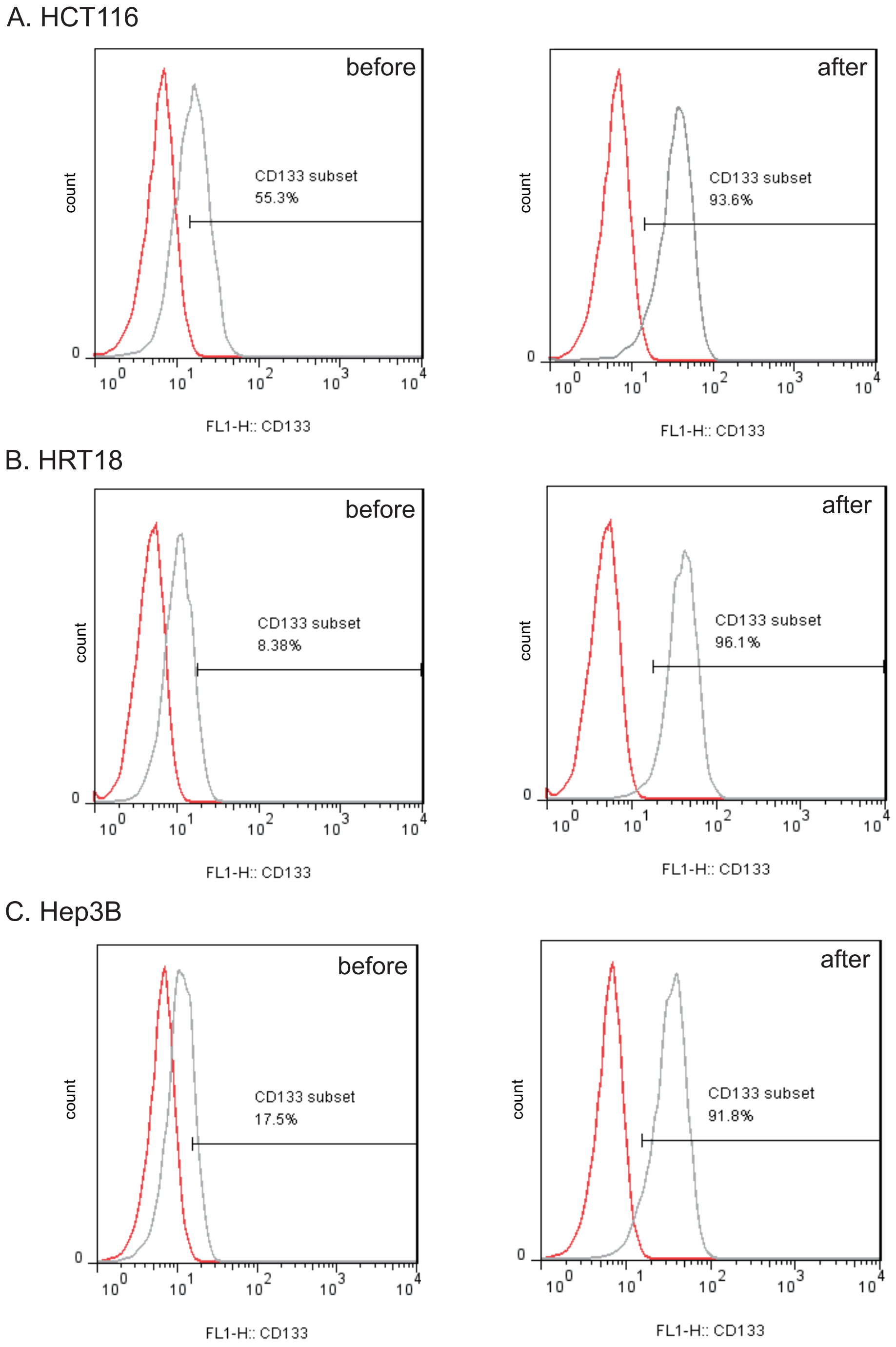

Supplementary Figure S1: Representative FACS of CD133 positive cells plots before and after sorting. $CD133^+$ cells were quantitated by FACS HCT116 **A.**, HRT18 **B.**, and Hep3B **C.** cells.

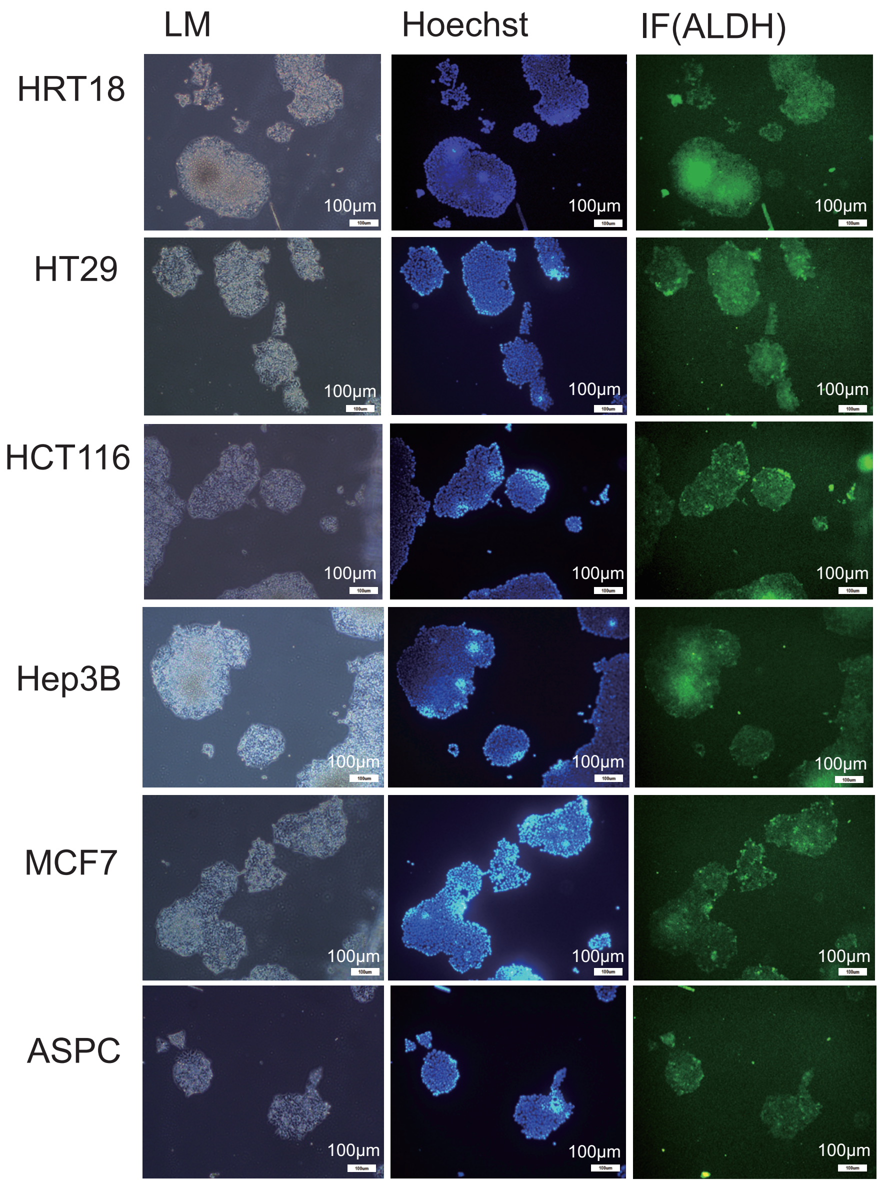

Supplementary Figure S2: Immunofluorescence (IF) staining of CSCs with anti-ALDH antibody. The sphere cells were treated with 4% paraformaldehyde, incubated with anti-ALDH antibody, and followed by the secondary antibody conjugated to fluorescent phycobiliproteins. Hoechst 33258 was used for nuclear counterstaining.

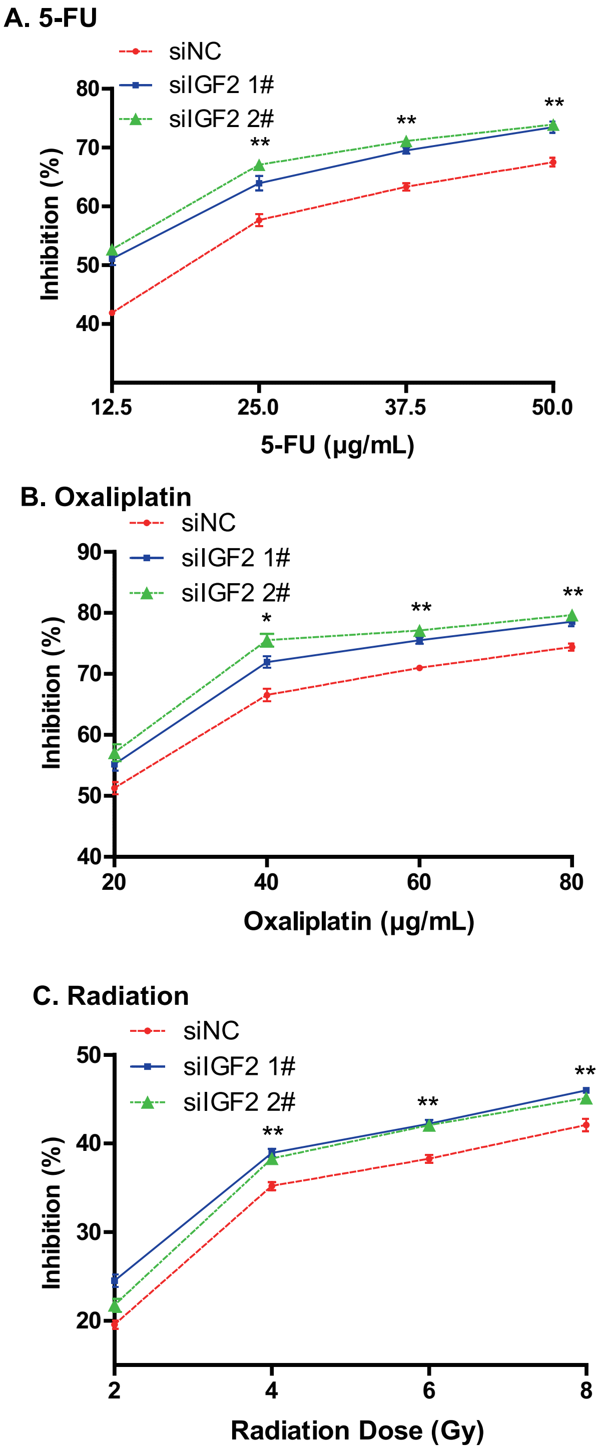

Supplementary Figure S3: Knockdown of *IGF2* increases cell sensitivity to chemo/radiotherapies in HCT116 CSCs. **A.** 5-FU treatment. HCT116 CSCs were treated with 5-FU (12.5, 25, 37.5, 50 μg/ml), respectively, for 72h and were evaluated by WST-1 cell proliferation assays. The *IGF2*-knockdown CSCs are more sensitive to 5-FU than the siNC-treated CSCs. $^{**}P<0.01$ as compared with control cells (siNC). **B.** Oxalipatin treatment. CSCs were treated with oxalipatin (0, 20, 40, 80, 160, 320 μg/ml). $^{*}P<0.05$ and $^{**}P<0.01$ as compared with control cells (siNC). **C.** Radiotherapy. HCT116 CSCs were irradiated with 2, 4, 6 and 8 Gy radiation, respectively. Cell proliferation was evaluated by WST-1 cell proliferation assays. *IGF2* knockdown sensitizes HCT116 CSCs to radiotherapy. $^{**}P<0.01$ as compared with control cells (siNC).

Supplementary Table S1: PCR oligonucleotide primers

Oligo Name	ID	Oligo sequence
PCR primers		
Apa1	J923	CTTGGACTTTGAGT CAAATTGGCCT
Apa1	J924	GAGGAGCCAGTCTGGGTTGTTGCTA
Alu1	2949	GTCCCCTCCTCTGCCATCACCTGA
Alu1	2950	GGATTTTGCCGGAAATATTAGCGT
ACTB	J880	CAGGTCATCACCATTGGCAATGAGC
ACTB	J881	CGGATGTCCACGTCACACTTCATGA
3C primers		
Exon4 1st	SJ37	CACTGAGTCATCTCAAAGTTAAGC
Exon4 2nd	SJ38	ccttcaagcacactgcacactcc
Exon5a 1st	SJ39	ctcct ggtggggcgc tgggagc
Exon5a 2nd	SJ40	tgcctgcccggagaccccagctc
Exon5b 1st	SJ41	tccttgatacaacagctgacctca
Exon5b 2nd	SJ42	ccgaaaagtacaacatctggcc
CTCF 1st	SJ43	cGGCCAATCAGAGCAGGGCCCTC
CTCF 2nd	SJ44	GCCCACCTCCGCCCTGGACAGT
CTCF 1st	SJ45	CACCCCCTCCCTCACCCTGCTCCT
CTCF 2nd	SJ46	CTAGCCCGGGCTTTTTCTAACTG
Histone methylation primers		
M1	T150	TCTGTCTCCTACGAAGTCCCCAGAG
M1	T151	GAAGCCCTCCCTGTCCACGTCCTGA
M2	T152	TGCCTGCCCGGAGACCCCAGCTCAC
M2	T153	CGCAGAGCGCCAAGGCCATGCTGAA
